# The library's role in countering infodemics

**DOI:** 10.5195/jmla.2021.1044

**Published:** 2021-01-01

**Authors:** Philip Walker

**Affiliations:** 1 philip.d.walker@vanderbilt.edu, Director, Annette and Irwin Eskind Family Biomedical Library and Learning Center, Vanderbilt University, Nashville, TN

## Abstract

Internet and communication technologies enable the creation of tremendous amounts of textual, graphic, and pictorial information. User-generated content published through personal web pages, blogs, and social media platforms has not only increased the amount of information available, but also expanded its reach. However, this ubiquity of information and empowerment of its creators leads to potentially controversial, futile, and inaccurate content circulating throughout the world. In the case of the COVID-19 pandemic, this can create false hope, fear, anxiety, harm, and confusion amongst information stakeholders. The World Health Organization recently applied the term “infodemic” to the COVID-19 pandemic. This commentary briefly discusses the current infodemic, its potential consequences, and the role of libraries—specifically health sciences, biomedical, and medical libraries—to help counter the COVID-19 infodemic. The discussion also has relevance for infodemics relating to other health and non-health affairs.

## INTRODUCTION

Current events in the Information Age generate a tremendous amount of textual, graphic, and pictorial information, which is disseminated through traditional broadcast television and radio channels, as well as Internet and communication technologies via the web. User-generated content published through personal web pages, blogs, and social media platforms has not only increased the amount of information available, but also expanded its reach. The ease of distributing information—whether by self-publishing, forwarding, or re-tweeting—renders it almost uncontrollable and nearly impossible to filter the credible from the not credible.

The World Health Organization (WHO) recently applied the term “infodemic” to the COVID-19 pandemic [1]. According to the Oxford English Dictionary, an infodemic is “an excessive amount of information about a problem that is typically unreliable, spreads rapidly, and makes a solution more difficult to achieve” [2]. This surfeit depicts the constant barrage of information generated by governments, researchers, news outlets, and the lay public. For example, the deluge of published peer-reviewed articles, preprints, and other forms of grey literature related to COVID-19 has been astounding. As of August 28, 2020, a simple search on COVID-19 yields 47,405 results in PubMed, 1,717 on bioRxiv.org, 6,368 on medRxiv.org, and more than 6.4 million in Google. While admirable, this publishing fervor causes concern because the accuracy of the information can be difficult to discern and it can create stress for health professionals who are constantly altering their practice based upon ever-changing guidance.

Contextually speaking, another phrase more specific to the health sciences environment is what Ioannidis et al. termed the “medical misinformation mess” [3]. This term focuses on the scientific research domain as opposed to the broader consumer health information perspective. The “mess” entails not only the amount of information, but also the quality of the information and the research that produced it, the inability of many practitioners to evaluate the information, and the lack of access to credible information for patients and caregivers to make crucial decisions about their care [3]. This mess occurred before COVID-19, but this pandemic has created a market where journal editors are flooded with manuscripts to review and the public has an insatiable thirst for new information from news outlets and social media around the clock [4].

Consumers address their health information needs online in several ways: by going directly to a resource, by finding the information with search engines, or by accessing the various forms of social media [5]. Over time, an information seeker develops a familiarity with and trust of a particular resource that fits their needs. A key factor here is trust in the source and its content. However, consumers must also comprehend that information. The numerous issues relating to poor health literacy, electronic health literacy, and health numeracy are beyond this commentary but are extremely important in relation to the current pandemic and the underlying health conditions that impact the health outcomes of those infected. Chong et al. provide a good editorial on e-health literacy and COVID-19 [6].

The web and social media have numerous benefits for disseminating information [7–9]. However, the constant barrage of information can cause psychological distress and other negative effects [10, 11]. In the case of the COVID-19 pandemic, health information consumers may experience stress due to the incessant exposure to information, independent of the information's accuracy or credibility. Furthermore, recent publications have commented on how bad science is getting into good journals, and then, almost instantly, the scientific article turns into a news story that is widely distributed into other media outlets. This has the unfortunate potential to cause anxiety, harm, fear, and increased risk for error and death [3, 4, 12–14].

These information issues surrounding the current pandemic serve as a valuable lesson for future national and international events, whether health-related or not. What can the library do and how can it help to offset the negative effects of the current and future infodemics?

## THE LIBRARY

What can health sciences librarians in academic medical centers and hospitals do to combat infodemics? First and foremost, we must ensure our clinical staff have access to credible information and resources. In this time of uncertainty and nonstop information dissemination, practitioners must be informed with the appropriate evidence to guide practice and decision making [3, 15].

While librarians cannot directly care for patients, it is comforting to know that we can support our clinical staff and help them reduce information overload and cognitive fatigue [12]. At the Eskind Biomedical Library, one of our major challenges was to conceive of approaches to efficiently deliver information to our practitioners and researchers, with the highest concern and need being for our nursing staff. Conversations with several nursing leaders motivated the health sciences librarians, which resulted in several activities:

1. With the assistance of our web team, we created a general “What's New with COVID-19” PubMed search string using PHP to automatically run the most recent search each day.
2. We created very specific premade searches that could be quickly accessed via a LibGuide.
3. We created and saved search alerts from PubMed and other literature databases.
4. We taught patrons how to create search alerts.
5. We taught patrons how to alter the aforementioned “What's New with COVID-19” PubMed search string.
6. We created and sent search summaries to clinical teams.
7. We created LibGuides with curated links to local, state, national, and international agencies and organizations.

During this pandemic, librarians have collaborated with our affiliated users to continue the missions of the research, education, and clinical enterprises. However, there is another group of stakeholders we must find ways to engage with: our nonaffiliated users. These users may vary from patients to the general public to other members of the health community. Regardless of their role, these groups may benefit from some of the previously mentioned projects. In our case, we quickly added COVID-19 links to our prominently placed Consumer Health Information LibGuide. Relationships with our nurse educators and patient advocacy groups helped to promote this valuable resource. However, in supporting these groups, it is important to remain cognizant of our licensing parameters to ensure that what is provided to the public is freely available or open access and to ensure content is written at the appropriate reading levels.

Why should academic medical center libraries or hospital libraries feel obligated to inform the public? First, we can serve as lighthouses or guides through the information deluge with the intention of allowing health sciences, biomedical, medical, school, and public libraries to use our content or link directly to it. Time, outreach, and collaboration are essential as we, the information professionals, work toward countering infodemics.

Second, recent studies by the Pew Research Center have strongly demonstrated the public's perception of public libraries as trustworthy, reliable, and helpful with learning new things and decision making [16]. I strongly believe health sciences and hospital libraries can serve a similar role by providing consumer health information resources on their home pages and promoting them to nearby public libraries as well as the numerous clinical educators and patient-family advocacy groups in our institutions. Studies continually show that opinions and research from scientists and health care providers are highly valued and trustworthy [17, 18]. We have the ability to broadly inform the citizenry, while helping to protect the reputation of the scientific and biomedical research domain and ensure the knowledge gained helps humanity, just as it did during past epidemics.

## CONCLUSION

This commentary is in no way inclusive of all the possible initiatives that libraries have participated in during the COVID-19 pandemic. It is solely focused on the barrage of information related to COVID-19 that has happened since January 2020. The Information Age has brought with it the great potential for the wide production and consumption of information. Unfortunately, information overload, information anxiety, misinformation and disinformation, rumors, and conspiracy theories abound, and they have consequences. The severity of this pandemic has created a critical time-sensitive demand for data and information directed toward clinicians, practitioners, consumers, patients, policymakers, and others. Not unique to this situation but a cause for concern is how this deluge of information impacts all aspects of society, because what is learned or implemented today may be based on information that may change tomorrow.

In this time of uncertainty and rapid information dissemination, it behooves librarians to explore opportunities to meet the information needs of core and new audiences and to remain focused on accessibility and readability when vetting information resources. The ability to address any infodemic depends upon our ability to identify and curate trustworthy, evidence-based knowledge resources that benefit all of the stakeholders involved in the pandemic information life cycle. Once relevant and appropriate information and resources are identified and organized by libraries, they need to be disseminated and promoted to users and referred to and linked throughout numerous information channels and platforms if we are to counter this and all future infodemics.
